# Black Phosphorus Based Field Effect Transistors with Simultaneously Achieved Near Ideal Subthreshold Swing and High Hole Mobility at Room Temperature

**DOI:** 10.1038/srep24920

**Published:** 2016-04-22

**Authors:** Xinke Liu, Kah-Wee Ang, Wenjie Yu, Jiazhu He, Xuewei Feng, Qiang Liu, He Jiang, Dan Tang, Jiao Wen, Youming Lu, Wenjun Liu, Peijiang Cao, Shun Han, Jing Wu, Wenjun Liu, Xi Wang, Deliang Zhu, Zhubing He

**Affiliations:** 1 College of Materials Science and Engineering, Shenzhen Key Laboratory of Special Functional Materials, Nanshan District Key Lab for Biopolymer and Safety Evaluation, Shenzhen University, 3688 Nanhai Ave, Shenzhen, 518060, People Republic of China; 2 Department of Electrical and Computer Engineering, National University of Singapore, 4 Engineering Drive 3, 117583, Singapore; 3 State Key Laboratory of Functional Materials for Informatics, Shanghai Institute of Microsystem and Information Technology, CAS, 865 Chang Ning Road, Shanghai, 200050, People Republic of China; 4 Department of Materials Science and Engineering, South University of Science and Technology of China, 1088 Xueyuan Road, Shenzhen, 518055, People Republic of China; 5 Institute of Materials research and Engineering (IMRE), 2 Fusionopolis Way, Innovis, #08-03, 138634, Singapore; 6 Department of Microelectronics, Fudan University, 220 Handan Road, Shanghai, 200433, People Republic of China

## Abstract

Black phosphorus (BP) has emerged as a promising two-dimensional (2D) material for next generation transistor applications due to its superior carrier transport properties. Among other issues, achieving reduced subthreshold swing and enhanced hole mobility simultaneously remains a challenge which requires careful optimization of the BP/gate oxide interface. Here, we report the realization of high performance BP transistors integrated with HfO_2_ high-*k* gate dielectric using a low temperature CMOS process. The fabricated devices were shown to demonstrate a near ideal subthreshold swing (*SS*) of ~69 mV/dec and a room temperature hole mobility of exceeding >400 cm^2^/Vs. These figure-of-merits are benchmarked to be the best-of-its-kind, which outperform previously reported BP transistors realized on traditional SiO_2_ gate dielectric. X-ray photoelectron spectroscopy (XPS) analysis further reveals the evidence of a more chemically stable BP when formed on HfO_2_ high-*k* as opposed to SiO_2_, which gives rise to a better interface quality that accounts for the *SS* and hole mobility improvement. These results unveil the potential of black phosphorus as an emerging channel material for future nanoelectronic device applications.

With the continuous shrinking of silicon field-effect transistors (FETs) over the past few decades, it has led to extraordinary improvement in the computation speed, functionality, and cost of the microprocessors^1^. However, the relentless scaling of gate length is approaching its fundamental scaling limit, which leads to increasing gate leakage. Undoubtedly, further transistor miniaturization has become increasingly challenging. In order to sustain Moore’s law, new materials (e.g. metal gate/high-k) and new device architectures (e.g. Fin structure, raised S/D etc) are required to be integrated in the silicon transistors, leading to improved device performance^2^,^3^,^4^. To enable further performance enhancement, the semiconductor industry has been actively exploring innovative approaches for enhancing the carrier transport in nanoscale transistors. This may be achieved via the adoption of new channel materials with superior carrier transport properties than traditional silicon^5^,^6^. However, attempts to use III–V compound semiconductors as replacement for silicon have seen very little success and still remain a very challenging task. With a bandgap larger than that of silicon and atomically thin geometry, 2-dimensional (2D) materials (MX_2_ e.g. MoS_2_, WSe_2_, etc)^7^,^8^,^9^,^10^,^11^ have an advantage for suppressing the source-to-drain tunneling current in ultra-scaled transistors and offers superior immunity to short-channel effects^12^. However, these semiconductors have relatively high effective mass and theoretical predication suggests that MX_2_ FETs may be better suited for low-power applications rather than high performance logic^13^.

Recently, several studies on black phosphorus (BP) FETs have been reported^14^,^15^,^16^,^17^,^18^,^19^,^20^,^21^. BP is the most stable form among the various allotropic modifications of phosphorus, and is a layered solid stacked with atomic layers via weak van der Waals interactions similar to graphite. Unlike MX_2_ semiconductor, BP is predicted to have a much lighter effective mass than that of MX_2_ (0.08–0.15 m_o_ along one of the in-plane directions) and the effective mass of BP is highly anisotropic with crystal orientation^22^. In addition, the bandgap of BP is expected to increase with decreasing layer thickness between ~0.3 eV and ~1.5 eV. However, monolayer phosphorene cannot be easily obtained via mechanical exfoliation, which could be due to the strong inter-layer coupling so that multilayer flakes are easier accessed^23^. Work done by H. Liu *et al*. in ref. 15 suggested that the higher hole mobility could be obtained using few-layer (FL) BP of thickness around 5 nm. To date, most of BP FETs were fabricated on exfoliated FL flake on SiO_2_/Si substrate, and exhibit large subthreshold swing *SS* in the range of 1.5–17.2 V/decade, which could be due to the poor interface quality between BP and SiO_2_^14^,^15^,^16^,^17^,^18^,^19^. Similar to introducing high-*k* materials into Si FETs, some preliminary work on the integration of high-*k* with BP has been carried out and the devices show some positive results, such as improved subthreshold swing^20^,^21^.

In this work, low temperature CMOS-compatible process was adopted to realize BP FETs which simultaneously achieved a near ideal sub-threshold swing and a high room temperature hole mobility using HfO_2_ as the gate dielectric and Nickel (Ni) as the source/drain electrodes. In addition, detailed material studies on FL and bulk BP have been carried out by low-temperature Raman spectroscopy and high-resolution x-ray photoelectron spectroscopy.

## Results and Discussion

**Figure 1:**
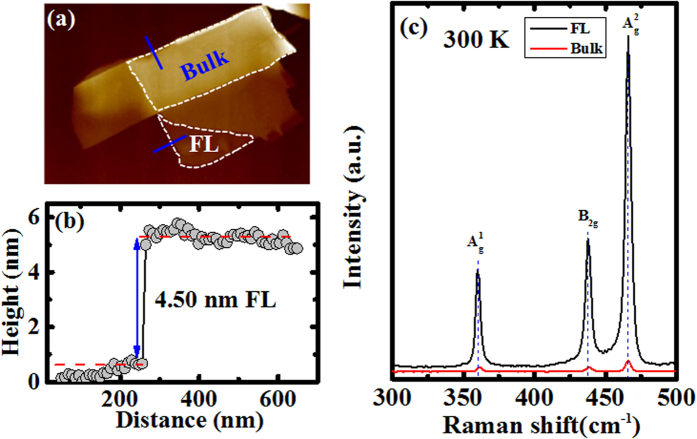
(**a**) The atomic force microscopy (AFM) image of the exfoliated BP flake, in which the few-layer (FL) and bulk BP layer are marked. (**b**) The film thickness was directly measured by AFM in a non-contact mode. The measured thickness is 4.50 and 41.78 nm, for FL and bulk BP film, respectively. (**c**) Raman spectra at 300 K of FL and bulk BP layer. Three active-Raman modes, 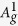, B_2g_, and 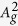 are clearly observed.

FL and bulk BP films are mechanically exfoliated on HfO_2_(5nm)/Si substrate, where the HfO_2_ layer was deposited by atomic layer deposition (ALD) tool. Atomic force microscopy (AFM) was used to directly measure the thickness of FL and bulk BP film, as shown in Fig. 1(a,b). The measured thickness is 4.50 and 41.78 nm, for FL and bulk BP film, respectively. According to the 0.85 nm monolayer thickness of BP, the layer number of FL and bulk BP is ~5 and ~45^23^. Figure 1(c) shows the Raman spectra of the FL and bulk BP film at 300 K using a 514 nm excitation laser. As compared to that of FL BP film, similar to MoS_2_ and graphene, bulk BP shows smaller intensity, which is due to the effect of optical interference^24^,^25^. The 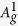, B_2g_, and 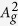 peaks can be well identified at Raman frequency of ~360.25, ~437.79, and ~465.99 cm^−1^, respectively, which is in good agreement with previously reported results^16^. Raman mode 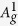 is related to the out-of-plane vibration of phosphorus atoms; Raman modes B_2g_ and 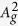 are associated to the in-plane vibration of phosphorus atoms, and the vibration directions of Raman modes B_2g_ and 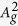 are in normal angle. As compared to the FL BP, the Raman modes 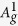 and B_2g_ of bulk BP have a slightly blue-shift of 0.75 and 0.58 cm^−1^, respectively. Similar to the MoS_2_, the out-of-plane Raman mode 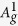 blue-shift of bulk BP is due to the increasing restoring force as the number of layer increases, or out-of-plane Raman mode 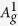 is stiffened with the increase of thickness because of the additional interlayer van der Waals interaction^26^. The shift in the frequency of the Raman mode 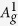 is consistent with the transition from few layers to bulk. As for the case of MoS_2_, in-plane Raman mode 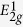 has a red-shift when the layer is increased, which is attributed to the dielectric screening mainly due to the presence of Mo atoms. In the case of BP, only phosphorus atoms are involved in vibration, dielectric screening should be negligible while the stacking induced structure changes may dominate^27^. As shown in Fig. 1(c), in-plane Raman mode B_2g_ of bulk BP have a slightly blue-shift of 0.80 cm^−1^, and in-plane Raman mode 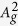 of bulk BP remains almost the same, when the thickness is increased to bulk. This could be due to the unique anisotropic structure of BP with different lattice parameters in various directions, which have different sensitivity to the external impact. Theoretical calculations show the lattice parameter along the out-of-plane direction changes significantly from bulk to few-layer BP, while the one in other two directions remain almost unchanged, which could be used to explain the anomalous vibration behaviors of BP^28^. It has been reported in ref. 15 that the 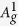 and 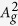 Raman modes shifts toward each other with increasing thickness due to the double resonance scattering can be the spectral fingerprint of identifying the single- and few-layer nature of the BP. In ref. 29 by J. L. Dattatray, the 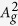 Raman mode of BP has a blue-shift of 1.6 cm^−1^ as the thickness decreasing, but B_2g_ and 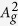 Raman modes of BP remain unchanged. It is noted that the BP samples in refs 15,29 are exfoliated on SiO_2_/Si substrate. It is speculated that the underlying substrate (SiO_2_ or HfO_2_) does affect the Raman peak position. In addition, the FL or monolayer BP was reported to be very sensitive to the ambient conditions, such as water and oxygen, as similar to the graphene and other 2D materials.

**Figure 2:**
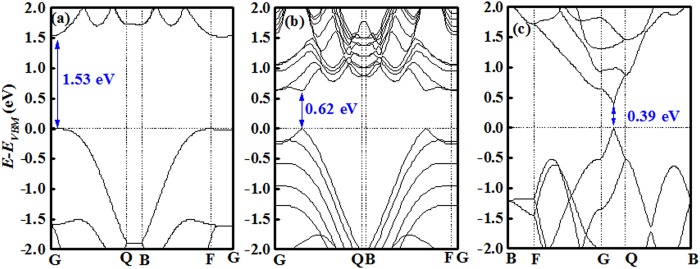
DFT-HSE06 band structure of (a) monolayer, (b) five layer, and (c) bulk BP film. The observed direct band gap is marked by arrow.

The band structures for monolayer, FL, and bulk BP are calculated using *ab initio* density function theory (DFT) with hybrid density functional HSE06. The generalized gradient approximation in the Perdew, Burke, and Ernzerhof (PBE) with ultrasoft pseudopotentials was used in the calculation of geometrical structure optimization process. For the calculation of monolayer and five-layer systems, we cut out <0 1 0> plan of bulk BP and configured 20 Å thickness of vacuum layer at c-axis, using 3 × 4 × 1 and 6 × 8 × 1 *k*-point grids for structural relaxation and band structure, respectively. The results are shown in Fig. 2(a–c). The direct bandgap value for monolayer, FL, and bulk BP is 1.53, 0.62, and 0.39 eV, respectively. The minimum conductance band and maximum valence band point is shifted from G point to the point located between G and Q, as the thickness increasing. The calculated effective hole mass for monolayer and five layer BP is ~6.3 m_o_ and 0.87 m_o_, respectively, which are in a good agreement with previous calculation results^28^. Lower effective hole mass, as compared to other 2D materials, can contribute to higher drain current and faster switching speed.

**Figure 3:**
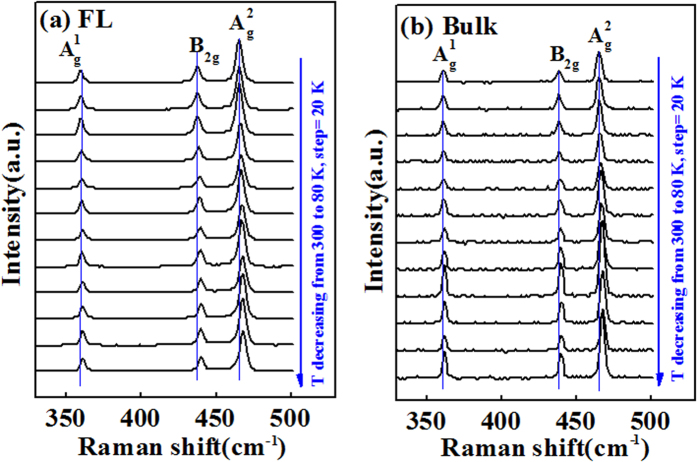
Temperature-dependent Raman spectra of (**a**) FL/HfO_2_ and (**b**) bulk BP/HfO_2_ samples at 80–300 K under a 514 nm excitation laser.

**Figure 4:**
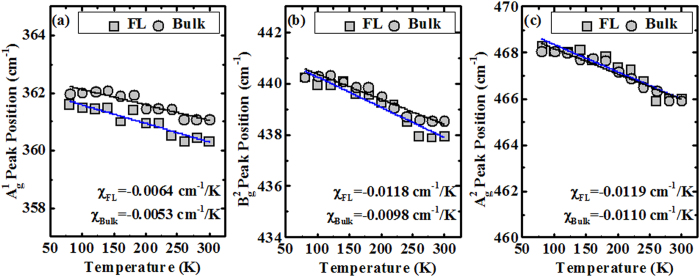
Effect of temperature variation on the Raman modes of (**a**) 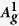, (**b**) B_2g_, and (**c**) 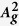 for FL and bulk BP film. With the decreasing temperature, the Raman mode is stiffened or shifted to higher frequency.

In order to differentiate this work with reported work in ref. 29 (BP/SiO_2_) and investigate the effect of underlying high-*k* HfO_2_ on BP Raman signal, temperature-dependent Raman measurements of FL/HfO_2_ and bulk BP/HfO_2_ samples have been carried out at 80–300 K under a 514 nm excitation laser, and the results are shown in Fig. 3(a,b), respectively. In this part, we will focus on the discussion about peak position as a function of temperature for FL and bulk BP. In view of BP as the potential CMOS channel material beyond Si, it is important to study the electron-phonon interactions or vibration modes under various temperatures through non-destructive Raman method. The temperature-dependent Raman vibration modes of BP can have a direct bearing on the carrier transport of BP-based FETs. When the temperature is decreased from 300 to 80 K, all the Raman modes of 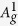, B_2g_, and 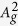 for FL and bulk BP film change linearly as a function of temperature, as shown in Fig. 4(a–c). It is well-known that Raman spectroscopy is a four-phonon process which is dominant over thermal expansion, as well as the phonon process on the Raman mode linearly shifts with change in temperature. A few data point dispersion for the Raman peak position can be expected and is well-understood due to the slight variation in the laser spot on the sample, or the local Raman stage vibration, or low excitation power on the sample followed by the extra attenuation from the cold-hot cell window during the measurement. The observed data of peak position obtained from Lorentzian fitting for 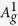, B_2g_, and 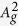 Raman modes versus temperature were fitted using the Grüneisen model: ω(T) = ω_0_ +*Χ*T, where ω_0_ is the Raman mode peak position at zero Kelvin temperature, and *Χ* is the first-order temperature coefficient of the same mode. The slope of fitted lines gives the first-order temperature coefficient of the specific Raman mode, and shown as an inset in Fig. 4. By rounding up *X* to the nearest two decimal points, the 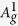, B_2g_, and 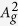 Raman modes show a *X* around −0.01 cm^−1^/K for both FL and bulk BP samples. Although the thermal coefficient (*Χ*) corresponding to 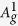, B_2g_, and 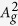 Raman modes of bulk BP has not been reported in the literature, the *Χ* values of these Raman modes for the FL BP (5 layers) is found to be comparable to the reported values (~−0.01 cm^−1^/K) in ref. 29. This indicates that the crystal structure of BP on HfO_2_ remains intact and is comparable to BP on SiO_2_. This is crucial to enable the realization of high performance device. The *Χ* obtained in this work is also similar to the one obtained for monolayer and bulk of MoS_2_ grown by chemical vapor deposition (CVD) or exfoliation in refs 30,31, and about one order larger than the one obtained for exfoliated monolayer WS_2_^32^. As compared to WS_2_, both FL and bulk BP are much more sensitive to the temperature. This would be due to the fact that the BP has better mechanical flexibility, which originates from its unique puckered crystal structure. Furthermore, the variation in the Raman peak position as a function of temperature for FL and Bulk BP samples is attributed to the temperature effect that results in anharmonicity and thermal or volume expansion.

**Figure 5:**
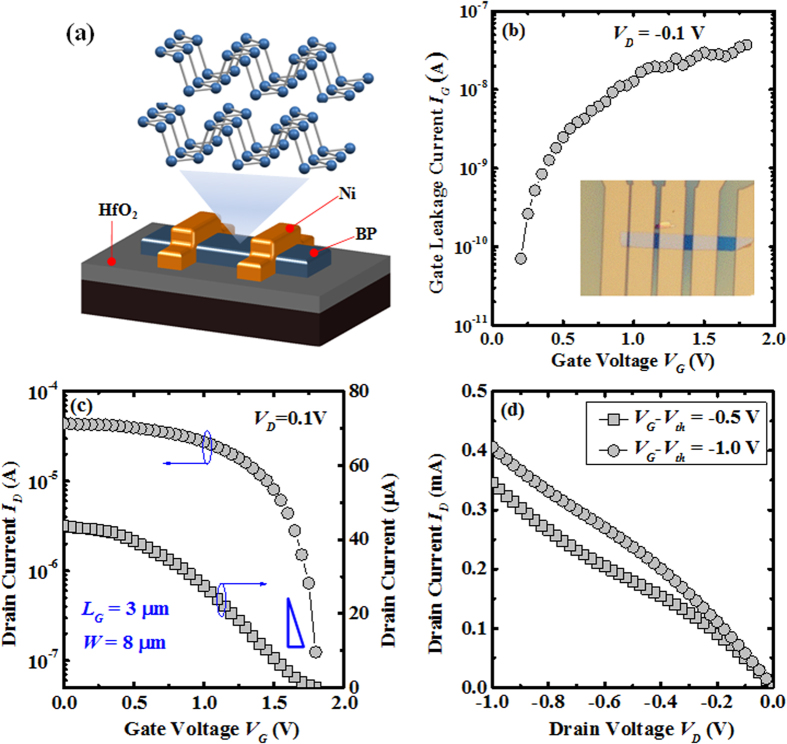
(**a**) Schematic drawing of fabricated BP FETs, and the inset shows the side view of BP film. (**b**) Gate leakage current as a function of gate voltage for the fabricated BP FETs, and inset shows the top-view of the fabricated BP FETs. The gate leakage current is in the range of 10^−8^~10^−10^ A under a drain voltage of −0.1 V in the measured gate voltage range. (**c**) Linear- and log-scale drain current as a function of gate voltage for the fabricated BP FETs with a gate length of 3 μm and a gate width of 8 μm. The gate voltage was swept from 0 V to positive voltage. Low hysteresis was obtained in this work, which further verifies the achievement of good BP/HfO_2_ interface quality as supported by the near-ideal subthreshold swing. The device shows on/off current ratio of ~10^2^. (**d**) Output characteristics (*I*_*D*_-*V*_*D*_) of fabricated BP FETs. The output drain current is about 0.4 mA under drain voltage of −1 V and gate-over-drive −1.0 V.

**Figure 6:**
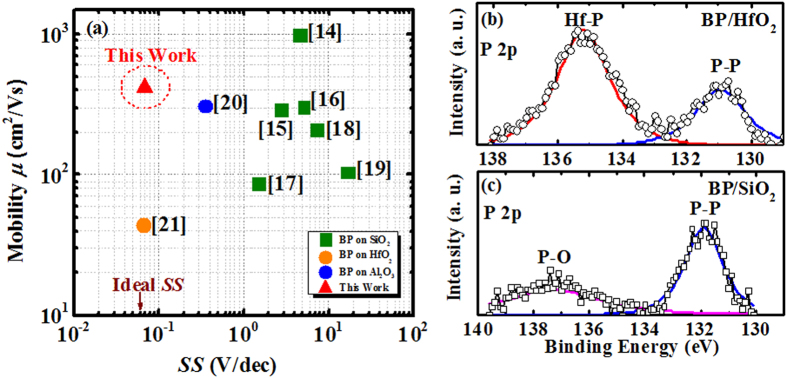
(**a**) A benchmark of the room-temperature hole mobility performance as a function of *SS* between this work and recently reported ones. This work simultaneously achieves a high room temperature hole mobility and near ideal subthreshold swing. P 2p XPS spectra of (**b**) BP/HfO_2_ and (**c**) BP/SiO_2_ samples. P-O bonding signal in BP/HfO_2_ interface is suppressed by the Hf-P bonding.

Figure 5(a) shows the device structure of BP FETs which have been fabricated on HfO_2_/Si substrate. The side-view of BP layer is shown as the inset of Fig. 5(a). The top-view of the fabricated devices is shown as the inset of Fig. 5(b). The fabricated devices with a gate length *L* of 3 μm and a gate width *W* of 8 μm were electrically measured. As shown in Fig. 5(b), the gate leakage current *I*_*G*_ is in the range of 10^−8^~10^−10^ A under a drain voltage of −0.1 V in the measured gate voltage range. As shown in Fig. 5(c), the fabricated BP FETs exhibit an on/off current ratio of ~10^2^ and a near-ideal subthreshold swing *SS* of ~69 mV/decade. The output drain current in this work is limited by the high contact resistance which could be further enhanced using source/drain engineering or doping technique. A threshold voltage *V*_*th*_ of ~1.7 V was extracted using the linear-extrapolation method, which extrapolates the (*I*_*D*_-*V*_*G*_) characteristic measured at *V*_*D*_ = 0.1 V, from the point of maximum slope to the intercept with the gate voltage axis. The effective interface state density *D*_*it*_ can be estimated by the equation of subthreshold swing *SS*: 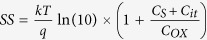, where *k* is the Boltzmann constant, *T* is the temperature in Kelvin, *q* is the electronic charge, 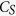 is the depletion capacitance of BP, 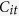 is the BP/HfO_2_ interface state capacitance, and 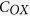 is the unit gate capacitance of 0.044 F/m^2^ (5 nm HfO_2_). When the applied gate voltage is near to the threshold voltage, 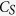 is the negligible compared to 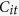, and then the effective interface state density *D*_*it*_ at BP/HfO_2_ interface can be estimated using the following equation: 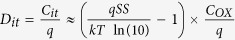. Based on the extracted *SS* of ~69 mV/decade, the effective interface state density *D*_*it*_ at the BP/HfO_2_ interface is calculated to be 4.38 × 10^12^ cm^−2^ eV^−1^. The interface states could be related to the dangling bonds due to the formation of phosphorus vacancies at the BP/HfO_2_ interface. Comparing to other 2D material such as MoS_2_^33^, the density of point defects (sulfur vacancies) has been reported to be 1.2 × 10^13^ cm^−2^, which is higher than that achieved in this work. A peak hole field effect mobility *μ* of ~413 cm^2^/V.s at 300 K can be extracted using 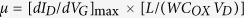, where *C*_*OX*_ is 0.044 F/m^2^ (dielectric constant HfO_2_ of 25) and *V*_*D*_ = 0.1 V. The high room temperature hole mobility achieved in this work is attributed to the better BP/HfO_2_ interface quality. This is supported by the XPS results as shown in Fig. 6(b,c) where P-O bonds are replaced by P-Hf bonds. Good BP/HfO_2_ interface quality, in term of low interface state density and suppression of P-O bonds, is the primary factor contributing to the good mobility achieved in this work. In Fig. 5(d), the output current of fabricated BP FETs is about 0.4 mA under a drain voltage of −1 V and a gate-over-drive of −1.0 V. A figure of merits shown in Fig. 6(a) benchmarks the room-temperature hole mobility performance as a function of *SS* between this work and recently reported work. The highest hole mobility *μ* of ~1000 cm^2^/V.s was obtained in ref. 14 on SiO_2_/Si substrate, but the *SS* is ~4.6 V/decade, which is too high to be practical for device application. In general, *SS* of BP FETs fabricated on SiO_2_/Si substrate is in the range of 1.5–17.2 V/decade, which is due to the poor interface quality located at BP/SiO_2_ interface. With an incorporation of high-*k* material (Al_2_O_3_, HfO_2_, etc) as the gate dielectric, the *SS* of BP FETs could be further reduced down to near-ideal value (~60 mV/decade), which indicates that better interface quality could be obtained for BP/high-*k* interface. As compared to the reported mobility (0.1~368 cm^2^/V.s) of MoS_2_ and WS_2_ FETs^8^,^34^, the mobility of BP FETs is significantly higher, this could be due to the lower effective mass of BP and better interface quality of BP/oxide, which explicates the advantage of BP over other 2D materials in the electronic application^28^. Also, the presence of high-*k* dielectric (HfO_2_) for BP FETs in this work can enhance the carrier mobility due to charging screening effect, which has also been observed in other 2D material (MoS_2_ etc) based devices^35^. Also, the carrier mobility carrier mobility in phosphorene is mainly limited by remote charge impurities, not phonon scattering^36^. Lower interface state density in this work can lower down charges interface state scattering, which results in mobility enhancement. In this work, both high room temperature hole mobility and near-ideal *SS* are simultaneously obtained for BP FETs on HfO_2_/Si substrate using a low temperature CMOS compatible process. Further, high-resolution X-ray photoelectron spectroscopy (XPS) is employed to study the interface chemical properties of BP/HfO_2_ and BP/SiO_2_ interface, as shown in Fig. 6(b,c). A board P-O peak (~137.22 eV) was observed on the BP/SiO_2_ sample, and was replaced by Hf-P peak (~135.22 eV) in BP/HfO_2_ sample. Also, the P-P peak for the BP/SiO_2_ sample is shifted to higher binding energy by 0.94 eV from 130.95 eV (BP/HfO_2_) to 131.89 eV (BP/SiO_2_). Based on P 2p XPS spectra, P-O bonding signal in BP/HfO_2_ interface is suppressed by the presence of Hf-P bonding in the BP/HfO_2_ sample, which implies that the BP is much more chemically stable on HfO_2_ surface, leading to high BP/HfO_2_ interface quality^35^. In other words, the high mobility performance achieved in this work is primarily attributed to the low interface state density and suppression of P-O bonds at the BP/HfO_2_ interface.

## Conclusions

Using low temperature CMOS-compatible process, this work demonstrated high performance BP transistors with a near ideal subthreshold swing (*SS*) and enhanced hole mobility (*μ*) via the integration of HfO_2_ high-*k* gate dielectric. Record figure-of-merits with *SS* ~69 mV/dec and room temperature *μ* >400 cm^2^/Vs were simultaneously achieved, which are attributed to the improvement of BP/HfO_2_ interface quality as evidenced by the suppression of P-O bonding as compared to that observed in BP/SiO_2_ interface. The use of high-*k* gate dielectric further allows the achievement of low gate leakage current in the order of 10^−8^~10^−10^ A. Our experimental findings could pave the way for the adoption of BP as a new channel material for next generation transistor applications.

## Methods

### Sample Preparation and Device Fabrication

Bulk BP crystal was purchased from 2D Semiconductor. 5 nm HfO_2_ gate dielectric was deposited on a highly doped blanket p-type silicon wafer, using tetrakis(ethylmethylamino) hafnium and H_2_O as precursors, by atomic layer deposition (ALD) at a temperature of 200 °C with a deposition rate of 0.70 nm/cycle. Before HfO_2_ deposition, the highly doped blanket p-type silicon wafer went through a native oxide removal step using a dilute HF (HF:H_2_O = 1:100). BP flake with a thickness of 15 nm was mechanically exfoliated on HfO_2_/Si substrate in a dry glove box. Once locating the FL BP sample based on the optical contrast and Raman measurement, electron beam resist poly(methyl methacrylate) PMMA was spin-coated to protect the flakes because of its fast degradation in ambient. Next, electron beam lithography was employed to pattern the source/drain electrodes, and 100 nm Nickel contact metal was deposited using a thermal evaporation. Finally, the remaining resist was removed by the acetone lift-off process. The highest temperature during the device fabrication is 200 °C in the HfO_2_ deposition step.

### Materials and Electrical Characterizations

Raman spectra were collected in a Renishaw inVia confocal system in the backscattering configuration. The wavelength of the laser was 514.5 nm (2.41 eV) from an argon ion laser, the grating of 2400 grooves mm^−1^ was used to obtain more details of line shapes of the Raman band. The laser power on the sample was set at around 1.0 μw to avoid laser induced heating. The application of a 100x objective lens with a numerical aperture of 0.9 can provide us a spot size of ~1 μm, and spectral resolution was 1 cm^−1^. The Si peak at 520 cm^−1^ was used as a reference for wavelength calibration. Atomic force microscopy (AFM) images were obtained under tapping mode using Bruker Dimension Icon. All the electronic measurements were performed at room temperature using Keithley 4200 semiconductor analyzer. XPS spectra were obtained using VG ESCALAB 220i-XL system with a mono-chromatized Al Kα (1486.6 eV) x-ray source (a constant pass energy of 20 eV)^37^.

## Additional Information

**How to cite this article**: Liu, X. *et al*. Black Phosphorus Based Field Effect Transistors with Simultaneously Achieved Near Ideal Subthreshold Swing and High Hole Mobility at Room Temperature. *Sci. Rep.* **6**, 24920; doi: 10.1038/srep24920 (2016).
